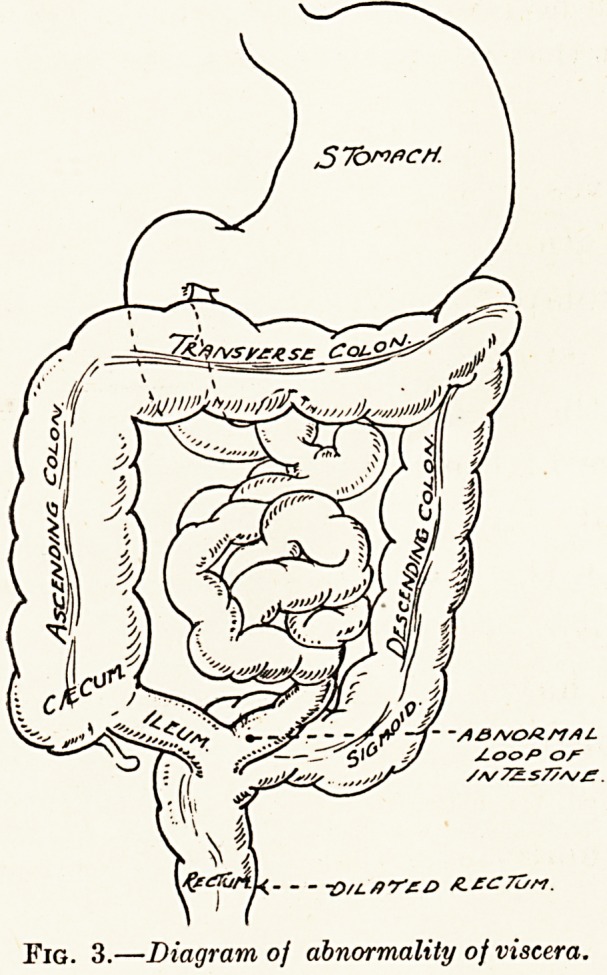# A Case of Rectal Prolapse

**Published:** 1929

**Authors:** D. Robertson, D. G. C. Tasker


PLATE IX.

FIG. 1.
*Clinical condition before operation.*

### A CASE OF RECTAL PROLAPSE.

BY

D. ROBERTSON, M.B., Ch.B. (Edin.),

AND

D. G. C. TASKER, M.S. (Lond.), F.R.C.S. (Eng.).

---

ON many occasions in the practice of surgery one is at a loss for an explanation of some pathological condition. One of the most bewildering of these occasions was furnished by a case of rectal prolapse.

When seen in March, 1927, the patient, an unmarried woman aged 40, complained of having something " come down from the back passage." On getting her to lie on her left side and inspecting the anus, the condition depicted in Fig. 1 presented itself. This extraordinary T-shaped protrusion became more inexplicable the more it was examined. Thus the examining finger could be passed into the anus quite easily around the stalk of the prolapse, the stalk appearing to come from part of the bowel higher up than could be reached with the finger. The protrusion itself was obvious bowel, covered with mucous membrane and turned inside out. Also the ends of the cross-piece of the T were open, and the fingers passed into each met in the middle and could both be passed up the stalk of the T. Vaginal examination revealed nothing, and abdominal examination was negative. By manipulation the whole prolapse could be reduced, so that ultimately nothing abnormal was palpable by a finger in the rectum or vagina.

Mere inspection and palpation of the prolapse did not suggest its nature, nor, as will be seen, did the following history that she gave us.

The medical history of her life was lengthy, and her description of past illnesses and of her symptoms was given with that meticulous care and accuracy so typical of a neurotic patient. Out of a great mass of details the following points appeared to be the salient features :—

As far as she could remember she had never been well in her life, and she had been in various institutions in Bristol on account of hemorrhages which appeared spontaneously in all parts of the body, but usually subcutaneously. The cause of these was ascribed to a blood disease—thrombocytopænia—but the only obvious finding in a complete blood count was an anæmia with 3,500,000 red blood cells to the cubic millimetre.

She had apparently always had some abdominal pain not typical of any organic condition, but had never had any marked exacerbation, nor had she ever had any operation. For some years she said she had had "piles," *i.e.* some bleeding from the rectum, and for the last eighteen months had had diarrhœa up to eight to ten times a day, with blood. For the last six months this prolapse had been coming down at infrequent intervals. During the last three weeks the prolapse had come down much more frequently, and was causing marked pain until it was reduced.

It was ascertained that she was not losing weight, and that she was able to take ordinary food, but for some reason she did not feel able to get about, but had remained in bed for some months. For some years she had been taking 4 oz. of laudanum a week to obtain sleep, although it did not appear that pain prevented this.

It was thought that the condition must be of the nature of some congenital diverticulum of the bowel which became prolapsed and everted, and it was hoped that X-ray examination with a barium enema and sigmoidoscopy would throw light on the cause of the prolapse. The sigmoidoscopy, however, showed nothing abnormal, and the barium enema showed a confusing mass of coils of intestine apparently doubled over on themselves.

It was thought that if the coming down of the prolapse could be prevented relief would be attained, and so in June, 1927, after two X-ray treatments to the spleen for the sake of the blood disease, an operation was performed under general anæsthesia. At this operation the colon appeared normal but voluminous, and the whole length of it was found mobile. No diverticulum was seen. The lower part of the sigmoid colon was fixed to the tissue on the ventral aspect of the iliac crest by turning back a small flap of peritoneum of the area and also a flap of peritoneum of the colon and suturing the two bare areas together. The abdomen was then closed and the patient put in the lithotomy position.

An incision was made between the coccyx and the anus, and by blunt dissection the area between the hollow of the sacrum and the rectum was opened up. This was packed with a large mass of gauze, which later was changed every other day.

The patient recovered well from this operation, and after two months the perineal incision was healed. For the first few days the bowels were kept closed with pulv. cretæ aromat. cum opio, but after this time the diarrhœa returned, at first about nine times a day and later as much as fifteen to twenty times a day.

All went well with the prolapse until July, 1928, when it reappeared, just the same as before, on two occasions. When it was reduced, digital examination whilst the patient was straining did not show any tendency to prolapse of the lower part of the rectum, and it was thought that the prolapsed piece of bowel was that between the part fixed to the side wall of the pelvis and the rectum fixed by the fibrous tissue resulting from the perineal operation.

The return of the prolapse had a marked mental effect on the patient, and she begged for something further to be done, and so in September, 1928, a further operation was performed. At this operation it was determined to fix in some way that part of the colon and rectum between the colopexy and the peritoneal floor of the pelvis, as it was felt that the secret of the prolapse lay there. This secret was now revealed. As will be seen from Fig. 2, which represents the state of things seen with the patient in the Trendelenburg position, there was a wide fistulous communication between a part of the lower ileum about two feet from the ileo-cæcal valve and the rectum immediately above the peritoneal floor. This communication was easily dealt with. It was divided between two clamps, and the gap in each viscus was closed with two layers of stitches, the abormal communication between ileum and rectum being thus abolished. No other abnormality was found, and it was seen that the fixation of the colon to the iliac fossa was still intact. Recovery from the operation was uneventful, but now instead of an intractable diarrhœa a most intractable constipation had supervened. Instead of fifteen to twenty actions of the bowels a day, there is such a constipation that in spite of prodigious doses of aperients no action of the bowels can be obtained except once in five days, and that only with repeated enemata. Without aperients or enemata it appears that the bowels would never be open of themselves. In March, 1929, there has been no return of the prolapse, and the patient is as before, except that the diarrhœa has been replaced by marked constipation.

Fig. 3 gives a diagram of the condition found at the second operation, and it will be readily appreciated that the prolapse consisted of the ileum prolapsing through the fistulous communication between ileum and rectum and then becoming like two intussusceptions.

The cause of the fistulous communication can only be guessed at. It did not appear to be congenital, as the union of the two pieces of bowel when divided appeared to consist of ordinary fibrous tissue. It seems that it must have originated from something in either the rectum or ileum having ulcerated through from one piece of bowel to the other. Whether there was some indigestible material swallowed by the patient which

FIG. 2.—*Condition discovered at operation. (Patient in Trendelenburg position.)*

FIG. 3.—*Diagram of abnormality of viscera.*

became impacted in the innermost part of the ileum, *i.e.* two feet from the ileo-cæcal valve, and then ulcerated through the adjacent rectum, or whether the communication was made from below by a foreign body inserted by the patient into the rectum, remains guess-work. The case, however, remains one of the strangest that we have been able to trace in surgical literature.

## Figures and Tables

**Fig. 1. f1:**
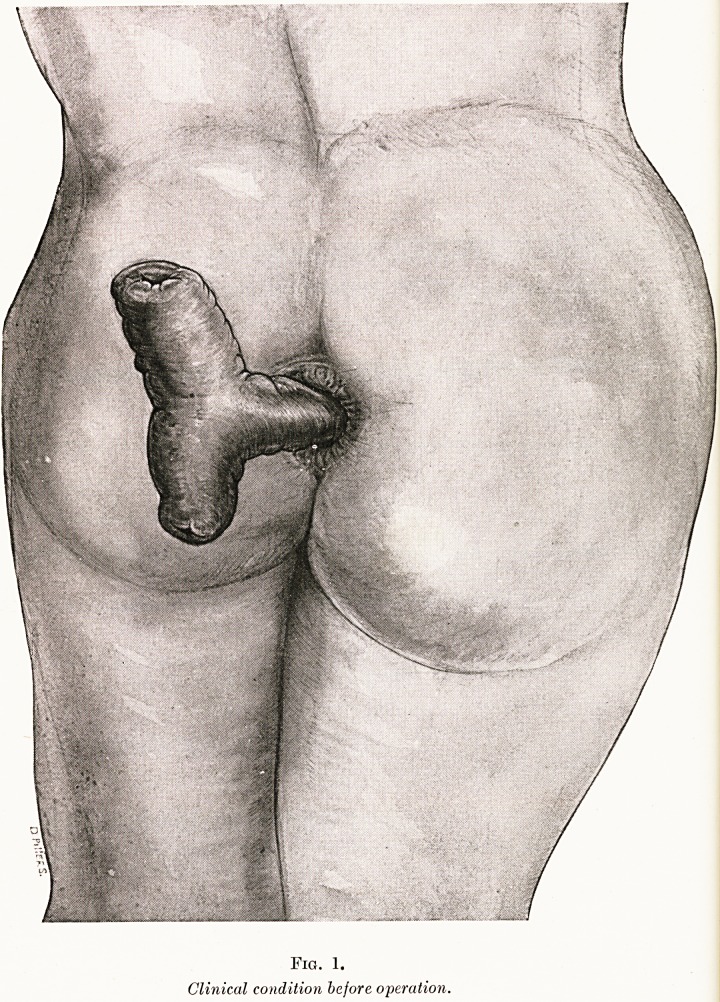


**Fig. 2. f2:**
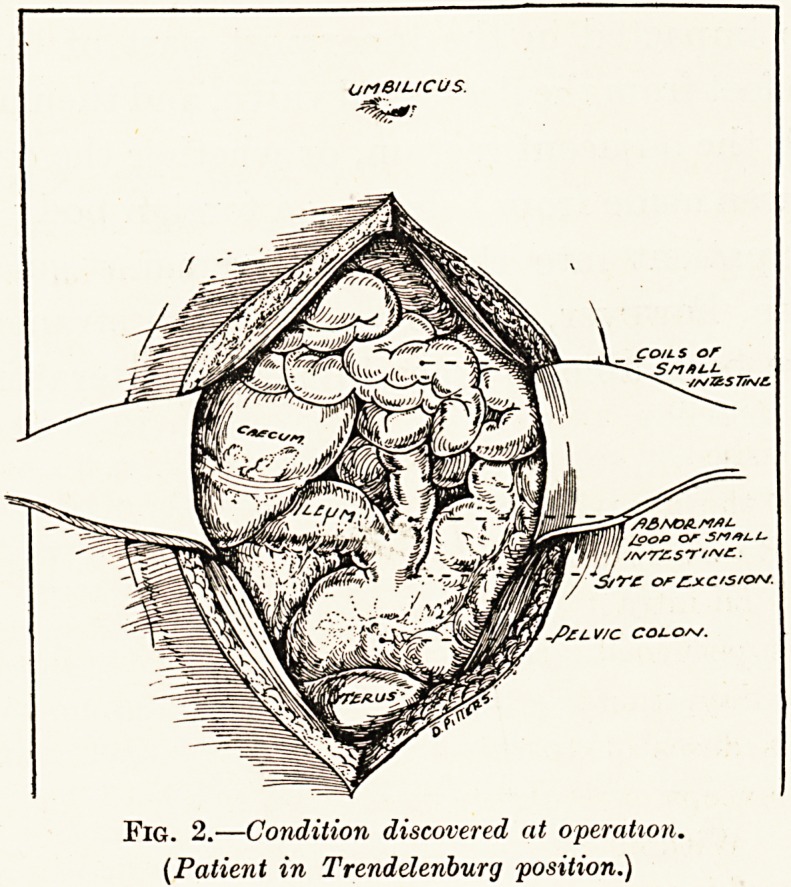


**Fig. 3. f3:**